# Successful Management of Ectopic Adrenocorticotropin-Secreting Thymic Carcinoid With Mitotane: A New Look at an Old Drug

**DOI:** 10.1210/jcemcr/luaf334

**Published:** 2026-02-27

**Authors:** Xiang Zhou, Yuan Xu, Xingtong Peng, Ruijie Hu, Lin Lu

**Affiliations:** Department of Endocrinology, Key Laboratory of Endocrinology of National Health Commission, Peking Union Medical College Hospital, Chinese Academy of Medical Sciences and Peking Union Medical College, Beijing 100730, China; Department of Thoracic Surgery, Peking Union Medical College Hospital, Chinese Academy of Medical Sciences and Peking Union Medical College, Beijing 100730, China; Department of Nuclear Medicine, Peking Union Medical College Hospital, Chinese Academy of Medical Sciences and Peking Union Medical College, Beijing 100730, China; Department of Endocrinology, Xi’an People's Hospital (Xi’an Fourth Hospital), Xi’an 710004, China; Department of Endocrinology, Key Laboratory of Endocrinology of National Health Commission, Peking Union Medical College Hospital, Chinese Academy of Medical Sciences and Peking Union Medical College, Beijing 100730, China

**Keywords:** ectopic adrenocorticotropin syndrome, Cushing syndrome, mitotane, thymic neuroendocrine tumor

## Abstract

Ectopic adrenocorticotropin syndrome (EAS) is usually associated with severe multiple complications and high mortality. Several adrenal steroidogenesis inhibitors can be used to control hypercortisolism when curative surgery is not feasible, but with different availability worldwide. It was long considered that mitotane (MTT) was not suitable for severe Cushing syndrome (CS) due to its delayed onset of action. We present a case of a 17-year-old girl with rapid-onset CS and an extremely high 24-hour urinary free cortisol (UFC) level (>300 times the upper limit of normal). An anterior mediastinal nodule with contrast enhancement was identified in computed tomography, with positive ^18^F-fluorodeoxyglucose (^18^F-FDG) positron emission tomography with computed tomography (PET/CT) uptake (maximum standardized uptake value = 10.1), suggestive of a thymic neuroendocrine tumor as the most likely cause of EAS. Preoperative MTT monotherapy titrated to 2 g/day reduced UFC by 85% within 13 days without adverse effects, stabilized severe neuropsychiatric disturbances and opportunistic infections, thus enabling successful thymectomy. The tumor turned out to be an adrenocorticotropin-secreting thymic typical carcinoid. Other EAS cases treated with MTT reported in the literature were reviewed, and the time needed to control hypercortisolism using MTT was shorter than previously reported. Instead of an “add-on drug,” we should reconsider the role of MTT in the treatment of severe hypercortisolism in EAS.

## Introduction

Cushing syndrome (CS) is a rare but serious endocrine disease resulting in chronic exposure to excess circulating glucocorticoids with multisystem effects. For pediatric patients, 98% are Cushing disease (CD) and adrenal-associated CS, and only 2% are ectopic adrenocorticotropin syndrome (EAS) [1]. EAS is usually associated with rapid progression of hypercortisolism, and the first-line treatment is surgical excision of adrenocorticotropic hormone (ACTH)-secreting neuroendocrine tumors (NETs). However, immediate curative surgery of the culprit tumor is not always possible.

In case of life-threatening CS, steroidogenesis inhibitors, used alone or in combination, are indicated to quickly control hypercortisolism. Fast-acting steroidogenesis inhibitors have been the first choice, such as metyrapone, ketoconazole, and osilodrostat. Mitotane (MTT) is less frequently used in severe CS due to its delayed onset of action and many side effects [2]. To compensate for its delayed action, MTT could be an “add-on drug” combined with fast-acting inhibitors [3]. Here we present a rare pediatric case of EAS caused by thymic NET treated with MTT monotherapy, with quick efficacy and no apparent side effects.

## Case Presentation

A 17-year-old girl was referred to our hospital for evaluation for progressive folliculitis and lower-limb edema for 3 weeks. Additionally, she developed moon face, abnormal menstrual cycles, and irritability for 3 months. Malignancy was remarkable in her family history, as both her grandmother and father had died from pancreatic cancer.

## Diagnostic Assessment

On physical examination, the patient’s height was 155 cm and her weight was 54 kg, with a body mass index 22.48 kg/m^2^ and waist circumference 81 cm. Blood pressure was 146/95 mm Hg. Multiple uniform erythematous brown papules were found on her face, neck, chest, back, and proximal upper limbs. She had moon face, supraclavicular fat depot and dorsocervical fat pad, but no cutaneous striae, skin thinning, bruising, proximal muscle weakness, or lower-limb edema.

Cortisol circadian rhythm was disrupted, with a morning plasma cortisol of 100 μg/dL (2760 nmol/L) (reference range [RR], 4.0-22.3 μg/dL, 110-615 nmol/L) and a midnight plasma cortisol of 66.9 μg/dL (1846 nmol/L). Morning plasma ACTH was 221.0 pg/mL (48.6 pmol/L) (RR, 7.2-63.3 pg/mL, 1.6-14.0 pmol/L). The mean 24-hour urinary free cortisol (UFC) was 23419.0 μg/24 hours (64636 nmol/24 hours) (RR, 13.2-77.2 μg/24 hours, 36.4-213.1 nmol/24 hours). The plasma cortisol after overnight 1-mg dexamethasone suppression test was 90.5 μg/dL (2498 nmol/L). Bilaterally inferior petrosal sinus sampling revealed no gradient between central and peripheral ACTH. Other laboratory tests results are shown in Table 1.

**Table 1.** Biochemical changes before and after mitotane treatment and thoracic surgery

	Reference range	Before mitotane	Mitotane d 3	Mitotane d 7	Mitotane d 11	POD 1	3-mo FU
24hUFC	13.2-77.2 μg/24 h	**23419 μg/24 h**	**12012.9 μg/24 h**	**6411.9 μg/24 h**	**3406 μg/24 h**	/	50.4 μg/24 h
	36.4-213.1 nmol/24 h	**64636 nmol/24 h**	**33156 nmol/24 h**	**17697 nmol/24 h**	**9400 nmol/24 h**	/	139 nmol/24 h
Cortisol	4.0-22.3 μg/dL	**105.4 μg/dL**	**90.8 μg/dL**	**53.7 μg/dL**	**48.8 μg/dL**	8.2 μg/dL	**22.6 μg/dL**
	110-615 nmol/L	**2909 nmol/L**	**2506 nmol/L**	**1482 nmol/L**	**1347 nmol/L**	226 nmol/L	**624 nmol/L**
ACTH	7.2-63.3 pg/mL	**221 pg/mL**	**242 pg/mL**	**203 pg/mL**	**201 pg/mL**	**4.8 pg/mL**	52.9 pg/mL
	1.6-14.0 pmol/L	**48.6 pmol/L**	**53.2 pmol/L**	**44.7 pmol/L**	**44.2 pmol/L**	**1.1 pmol/L**	11.6 pmol/L
HbA_1c_	4.5%-6.3%	6.20%	/	/	/	/	5%
	33.9-47.5 mmol/mol	46.7 mmol/mol	/	/	/	/	37.7 mmol/mol
Potassium	3.5-5.5 mmol/L	**3.1 mmol/L**	**3.3 mmol/L**	**3.4 mmol/L**	4 mmol/L	**2.9 mmol/L**	4.1 mmol/L
	3.5-5.5 mEq/L	**3.1 mEq/L**	**3.3 mEq/L**	**3.4** **mEq/L**	4 mEq/L	**2.9** **mEq/L**	4.1 mEq/L
Fasting blood glucose	3.9-6.1 mmol/L	5.6 mmol/L	4.2 mmol/L	5.9 mmol/L	4.6 mmol/L	4.5 mmol/L	4.8 mmol/L
	70-110 mg/dL	100.8 mg/dL	75.6 mg/dL	106.2 mg/dL	82.8 mg/dL	81.0 mg/dL	86.4 mg/dL
LDH	<250 U/L	**689 U/L**	**689 U/L**	/	**489 U/L**	**351 U/L**	185 U/L
TC	<5.2 mmol/L	3.43 mmol/L	3.07 mmol/L	/	3.52 mmol/L	/	3.59 mmol/L
	<201 mg/dL	132.6 mg/dL	118.7 mg/dL	/	136.1 mg/dL	/	138.8 mg/dL
TGs	<1.7 mmol/L	0.88 mmol/L	1.38 mmol/L	/	0.87 mmol/L	/	1.1 mmol/L
	<150 mg/dL	77.9 mg/dL	122.2 mg/dL	/	77.1 mg/dL	/	97.4 mg/dL
LDL-C	<3.4 mmol/L	2.03 mmol/L	1.86 mmol/L	/	2.32 mmol/L	/	1.26 mmol/L
	<131 mg/dL	78.5 mg/dL	71.9 mg/dL	/	89.7 mg/dL	/	48.7 mg/dL
HDL-C	>1.0 mmol/L	1.09 mmol/L	1.86 mmol/L	/	**0.96 mmol/L**	/	2.06 mmol/L
	>39 mg/dL	42.2 mg/dL	71.9 mg/dL	/	37.1 mg/dL	/	79.7 mg/dL
ALT	6-29 U/L	**77 U/L**	**55 U/L**	**46 U/L**	**64 U/L**	**45 U/L**	14 U/L
GGT	7-45 U/L	/	32 U/L	**52 U/L**	**54 U/L**	/	26 U/L
Testosterone	0.10-0.84 ng/mL	**1.23 ng/mL**	/	/	/	/	0.35 ng/mL
	0.35-2.91 nmol/L	**4.27 nmol/L**	/	/	/	/	1.21 nmol/L
Lymphocytes	1200-3800/μL	**630/μL**	/	/	**780/μL**	/	2400/μL
B cells	160-350/μL	197/μL	/	/	186/μL	/	**130/μL**
Natural killer cells	155-550/μL	**97/μL**	/	/	**125/μL**	/	**660/μL**
T cells	940-2140/μL	**312/μL**	/	/	**460/μL**	/	1567/μL
CD4^+^ T cells	550-1200/μL	**153/μL**	/	/	**184/μL**	/	881/μL
CD8^+^ T cells	380-790/μL	**140/μL**	/	/	**250/μL**	/	602/μL
CD4^+^/CD8^+^ ratio	0.9-2.0	1.09	/	/	0.74	/	1.46

Abnormal values are shown in bold font. “/” means “nonapplicable”.

Abbreviations: ACTH, adrenocorticotropic hormone; ALT, alanine transaminase; FU, follow-up; GGT, γ-glutamyl transpeptidase; HbA_1c_, glycated hemoglobin A_1c_; HDL-C, high-density lipoprotein cholesterol; LDH, lactate dehydrogenase; LDL-C, low-density lipoprotein cholesterol; POD, postoperative day; TC, total cholesterol; TGs, triglycerides; UFC, urinary free cortisol.

**Figure 1.**
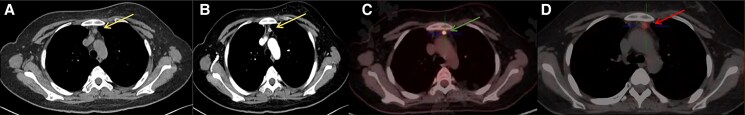
A and B, Contrast-enhanced chest computed tomography (CT); C, ^18^F-FDG-PET/CT; and D, ^68^Ga-DOTATATE-PET/CT scan. A 7-mm round nodule in the anterior mediastinum with marked enhancement (yellow arrow) in chest CT showed positive uptake in ^18^F-FDG-PET/CT (green arrow) and ^68^Ga-DOTATATE-PET/CT (red arrow), with maximum standardized uptake value 10.1 and 2.3, respectively.

Dynamic contrast-enhanced magnetic resonance imaging of the pituitary gland demonstrated a 3 × 6-mm hypoenhancing lesion in the left posterior lobe, raising the possibility of a microadenoma. Computed tomography (CT) demonstrated a 7-mm nodule in the anterior mediastinum with marked enhancement (Fig. 1A and 1B). The aforementioned nodule was negative on ^99m^Tc-HTOC octreoscan, but had intense uptake on ^18^F-fluorodeoxyglucose (^18^F-FDG) positron emission tomography CT (PET/CT) with a maximum standardized uptake value (SUVmax) 10.1 (Fig. 1C) and mild uptake in ^68^Gallium DOTA-(Tyr3)-octreotate (^68^Ga-DOTATATE) PET/CT with SUVmax 2.3 (Fig. 1D). EAS arising from thymic NET was highly suspected.

## Treatment

**Figure 2.**
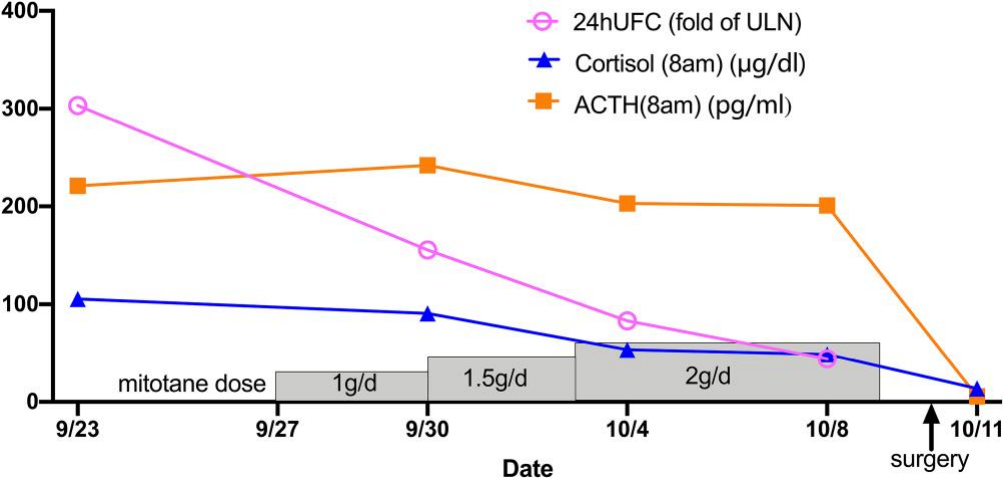
Change of cortisol, adrenocorticotropic hormone (ACTH), and 24-hour urinary free cortisol (24hUFC) perioperatively. 24hUFC decreased dramatically from 303-fold of the upper limit of normal (ULN) to 44-fold after using mitotane for 11 days. The patient achieved remission postoperative day 1, with cortisol (8 Am) and ACTH (8 Am) reaching 8.2 μg/dL and 4.8 pg/mL, respectively.

Spironolactone (40 mg/day) combined with potassium chloride sustained-release tablets (3.0 g/day) was administrated to correct the patient’s hypokalemia as well as to control hypertension. Metformin (2.0 g/day) was administrated to control postprandial hyperglycemia. Surgery was arranged by a thoracic surgeon immediately. However, the patient’s condition deteriorated abruptly. She became extremely irritable and psychotic, even rolling on the floor screaming in the ward. Concurrently, she was immunosuppressed with lymphocytopenia, and the count of CD4^+^ lymphocytes was as low as 153/μL (RR, 550-1200/μL), thus prophylactic sulfamethoxazole and trimethoprim (SMZ-TMP) was administered. Multiple infections were identified and treated accordingly: *Malassezia* folliculitis was treated with itraconazole, cytomegaloviremia was treated with ganciclovir, and urinary tract infection with *Klebsiella pneumoniae* and *Escherichia coli* was treated with ertapenem. A multidisciplinary team comprising endocrinologists, oncologists, urology experts, psychiatrists, and pharmacists was organized and decided on medication to control hypercortisolism rather than an urgent surgery. Other steroidogenesis inhibitors were not available, so MTT was chosen for off-label use. We started the dose of MTT at 1 g/day, and titrated to the maintenance dose 2 g/day by day 7. The patient’s 24hUFC decreased remarkably by 85% within 13 days (Fig. 2). Changes in other laboratory tests are provided in Table 1. Cytomegaloviremia and urinary culture turned negative, and her psychotic manifestations normalized. She underwent videothoracoscopic surgery the 17th day after admission, and a thymus with an 8-mm nodule was successfully removed. Histopathology was consistent with a well-differentiated typical carcinoid, with Ki-67 2% and fewer than 2 mitotic figures per 2 mm². Immunohistochemistry was positive for ACTH. No mutation was detected in the *MEN1* gene.

## Outcome and Follow-up

Postoperative hydrocortisone was started immediately with a stress dose (intravenous hydrocortisone 100 mg, every 8 hours) and gradually decreased to a maintenance dose. The patient’s antihypertensive and antiglycemic medication, as well as potassium supplements, were stopped. Her menstruation resumed 1 month after surgery. Hydrocortisone was discontinued 3 months later.

## Discussion

In our case, we highlight 2 points. First, thymic NET is a rare but important cause among pediatric EAS patients. Second, this patient responded well to MTT monotherapy, with good and fast efficacy and no apparent side effects.

EAS occurs rarely in pediatric patients, accounting for only 2.6% of the cases of ACTH-dependent CS in adolescents [1]. Similar to their adult counterparts, the predominant etiologies of pediatric EAS are well-differentiated NETs, with bronchial, thymic, and pancreatic NETs being listed as the leading etiologies, especially among children older than 8 years [1, 4]. In our case, thymic carcinoid was identified. Most thymic NETs occur in adulthood [5], and cases in the pediatric group are quite few. We searched for pediatric thymic EAS cases in the English literatures and identified only 17 other cases from 1991 to 2024. The median onset age was 13 years (range, 2.5-18 years), with no sex predominance. Cushingoid appearance (61%), hypokalemia (44%), and weight gain without concomitant height gain (38%) were the most common manifestations. A total of 22% patients exhibited neuropsychiatric and mood disorders, including emotional lability, depression, anxiety, and personality change. Both hypercortisolism and metabolic alkalosis might cause or contribute to the development of neuropsychiatric symptoms. The degree of elevation in ACTH and 24hUFC varied greatly. The median tumor size was 30 mm (range, 4-115 mm). Although x-ray and CT are capable of identifying large nodules or masses of 10 mm or greater, somatostatin receptor and ^18^F-FDG PET might be more sensitive in identifying smaller ectopic thymic lesions, as in the case reported by Aldhoon-Hainerová et al [6] and our case. Histology subtype was not described in detail in 6 cases, and in the remaining cases, 7 patients had an atypical carcinoid, 4 had a typical carcinoid, while 1 patient had an endocrine carcinoma with large cells. A total of 83% patients underwent thymectomy. Two patients had persistent active CS, and 4 patients had recurrence and/or metastasis after a median follow-up time of 30 months (range, 15-36 months). Seven patients died after a median follow-up time of 57 months (range, 5-120 months). Higher stage, poor histology subtype, and incomplete or no resection were predictors for worse overall survival [6]. For the present case, we achieved R0 resection, and the histology was typical carcinoid, which indicated a better outcome, but long-term follow-up is indicated.

To manage severe hypercortisolemia, preoperative MTT was administrated in this case, which was efficient and safe. MTT, approved for treatment of adrenocortical carcinoma, has been used to treat CD since 1960s [7]. It was long considered that MTT was not suitable for severe CS due to its delayed onset of action [2]. In the study by Baudry et al [8], which included 56 patients with proven CD as well as 20 patients with highly suspected CD, the median time for these patients to reach remission was 6.7 months. Therefore, in emergent life-threatening cases, MTT monotherapy is not recommended; instead, concomitant treatment with 2 or 3 adrenal steroidogenesis inhibitors were reported to achieve rapid control of hypercortisolism [3]. However, in our case, MTT monotherapy led to a rapid, striking decrease of 24hUFC by 85% after just 13 days of treatment, which allowed for dramatic improvement of neuropsychiatric disturbances and immunosuppressed state, and subsequently successful surgery. Our experience contrasts with previous perspectives [8], possibly due to different etiologies of CS, so we did a comprehensive literature review focusing on EAS patients.

Our search for publications on MTT and EAS yielded 11 relevant articles as of May 24, 2025, including 41 cases, with 31 cases treated with MTT monotherapy and 10 treated with multiple adrenal steroidogenesis inhibitors. Details are shown in Table 2 [3, 9-18]. MTT was administrated as a long-term therapy (>6 months) in 75.6% patients. Cortisol normalization was achieved in 85.4%. Among 14 patients with initial occult tumors, carcinoid was located during follow-up after initial MTT treatment in 11 cases. The largest cohort was from Donadille et al [10], in which 23 patients were treated with long-term use of MTT of 3.3 ± 1.2 g/d (range, 1.5-6 g/d) with good efficacy but delayed-onset effect. MTT effectively controlled hypercortisolism in all but 2 patients, decreasing UFC from 2603 ± 3443 μg/24 hours before treatment to 79 ± 169 μg/24 hours, but the time needed to achieve a normal UFC was as long as 130 ± 85 days (range, 16-307 days). However, Hu et al [9] recently reported a much shorter time to reach remission. Three patients with EAS caused by advanced pancreatic NETs were treated with MTT monotherapy at a maximal dose of 3 g/d, and their cortisol normalized within just 7 to 14 days. The severity of hypercortisolism in different cases might explain the efficacy variation, since the degree of hypercortisolism was more severe in our case. Adrenal hyperplasia was correlated with ACTH and 24hUFC levels. Patients with EAS usually have much higher ACTH, resulting in more striking increased adrenocortical activity. MTT appeared to suppress corticosteroid secretion more readily and more completely in patients with adrenal hyperfunction than in those with normal adrenal cortical function [7].

**Table 2.** Literature summary of mitotane therapy in patients with ectopic adrenocorticotropin syndrome

Case No.	Sex/Age, y	Diagnosis	Treatment	Maximal MTT dose, mg/d	Duration of MTT, mo	Cortisol change	Recovery time	Side effects	Follow-up, y	Outcome
1 [9]	M/40	Metastatic pNET	Surgery→OCT + CAPTEM→MTT	3	10	Normal	7 d	None	5.5	Died (tumor complications)
2 [9]	M/60	Metastatic pNET	CAPTEM→OCT + surufatinib + MTT	3	NA	Normal	6 d	Slight loss of appetite, dizziness	1.0	Died (tumor effects)
3 [9]	F/60	pNET	Surgery→CAPTEM + MTT	3	16	Normal	14 d	No	3.7	Died (tumor progression)
4 [10]	F/70	SCLC	MTT	NA	NA	Normal or low	NA	NA*^c^*	1.2	Died (tumor metastases)
5 [10]	F/60	SCLC	MTT	NA	NA	Normal or low	NA	NA*^c^*	1.4	Died (tumor metastases)
6 [10]	M/62	SCLC	MTT	NA	NA	Normal or low	NA	NA*^c^*	0.9	Died (tumor metastases)
7 [10]	F/66	SCLC	MTT	NA	NA	Normal or low	NA	NA*^c^*	0.6	Died (tumor metastases)
8 [10]	F/64	Metastatic ovarian cancer	MTT	NA	NA	Normal or low	NA	NA*^c^*	1.6	Died (tumor metastases)
9 [10]	M/50	Gastrinoma	MTT + tumor debulking	NA	NA	Normal or low	NA	NA*^c^*	0.4	Alive
10 [10]	F/65	Gastrinoma	MTT + tumor debulking	NA	2	Reduced but not normal	NA	Severe hepatoxicity*^c^*	3.3	Alive
11 [10]	F/60	Carcinoid	MTT + thyroidectomy	NA	<1	Reduced 85% but not normal	NA	NA*^c^*	4.4	Died (cardiovascular causes)
12 [10]	F/63	Carcinoid	MTT + lobectomy	NA	NA	Normal or low	NA	NA*^c^*	8.7	Died
13 [10]	F/50	Carcinoid	MTT + lobectomy	NA	NA	Normal or low	NA	NA*^c^*	2.8	Alive
14 [10]	M/56	Carcinoid*^a^*	MTT	NA	NA	Normal or low	NA	NA*^c^*	27.6	Died (cardiovascular causes)
15 [10]	F/44	Carcinoid*^a^*	MTT→lobectomy	NA	NA	Normal or low	NA	NA*^c^*	34.4	Died (unidentified cause in eucortisolic state)
16 [10]	F/42	Carcinoid*^a^*	MTT→lobectomy	NA	NA	Normal or low	NA	NA*^c^*	21.3	Alive
17 [10]	M/43	Carcinoid*^a^*	MTT→lobectomy	NA	NA	Normal or low	NA	NA*^c^*	11.9	Alive
18 [10]	F/32	Carcinoid*^a^*	MTT→lobectomy	NA	NA	Normal or low	NA	NA*^c^*	19.1	Alive
19 [10]	F/65	Carcinoid*^a^*	MTT→lobectomy	NA	NA	Normal or low	NA	NA*^c^*	6.4	Alive
20 [10]	F/43	Carcinoid*^a^*	MTT→lobectomy	NA	NA	Normal or low	NA	NA*^c^*	4.7	Alive
21 [10]	F/38	Carcinoid*^a^*	MTT→thymectomy	NA	NA	Normal or low	NA	NA*^c^*	16.7	Alive
22 [10]	F/80	NA	MTT	NA	NA	Normal or low	NA	NA*^c^*	0.4	Died (urinary sepsis)
23 [10]	F/54	NA	MTT	NA	NA	Normal or low	NA	NA*^c^*	3.3	Alive
24 [10]	F/34	NA	MTT	NA	NA	Normal or low	NA	NA*^c^*	12.4	Alive
25 [10]	M/35	NA	MTT	NA	NA	Normal or low	NA	NA*^c^*	1.3	Alive
26 [10]	F/58	NA	MTT	NA	NA	Normal or low	NA	NA*^c^*	3.8	Alive
27 [11]	M/41	Occult EAS	MTT		3	Very low	NA	No	1.8	Alive
28 [12]	NA	Bronchial carcinoid*^a^*	OCT + KTZ + MTT→BA→lobectomy	NA	NA	Not normal	NA	NA	NA	NA
29 [13]	F/40	Typical bronchial carcinoid tumor*^a^*	MTT→sella turcica raidotherapy→BA→open-chest surgery	12	9	Low	1 mo	No	15	Alive
30 [14]	M/42	Occult EAS	Hemihypophysectomy→KTZ→KTZ + MTP + MTT→BA	2	18	Low	NA	No	11	Alive
31 [15]	M/53	SCLC	chemotherapy→MTT	2.5	1.4	Reduced but not normal	NA	No	5	Died
32 [3]	F/65	Occult EAS	MTT + MTP + KTZ	3	19	Reduced but not normal*^b^*	1-3 d	No	19	Alive
33 [3]	F/29	Mediastinal carcinoid tumor*^a^*	MTT + MTP + KTZ→surgery	3	6	Normal	1-3 d	No	35	Alive
34 [3]	F/66	Metastatic thymic neuroendocrine carcinoma	MTT + MTP + KTZ	3	14	Normal	1-3 d	Dizziness, confusion, liver toxicity	14	Died (tumor progression)
36 [3]	F/75	Occult EAS	MTT + MTP + KTZ	3	9	Normal	1-3 d	NA	10	Died
37 [3]	73/M	Metastatic neuroendocrine lung cancer	MTT + MTP + KTZ	3	1	Reduced but not normal	NA	Liver toxicity	1	Died
38 [3]	46/M	Metastatic pancreatic neuroendocrine carcinoma	MTT + MTP + KTZ	3	4	Normal	1-3 d	NA	4	Died
39 [3]	39/F	Neuroendocrine tumor of small intestine	MTT + MTP + KTZ	3	1	Normal	1-3 d	NA	6	Alive
35 [18]	F/56	Metastatic melanoma	Chemotherapy→MTP + aminoglutethimide→MTT + MTP	4	4	Normal	NA	No	NA	NA
40 [17]	M/51	Bronchial carcinoid	Pituitary radiation→MTT→hypophysectom	4	3	Low	NA	No	12	Died (cardiogenic shock)
41 [18]	M/35	Thymic tumor	MTT→open-chest surgery	12	3	NA	NA	No	>0.25	Died (cardiac and respiratory failure)

Abbreviations: BA, bilateral adrenalectomy; CAPTEM, capecitabine and temozolomide; EAS, ectopic adrenocorticotropin syndrome; F, female; KTZ, ketoconazole; M, male; MTP, metyrapone; MTT, mitotane; NA, nonapplicable; OCT, octreotide; pNET, pancreatic neuroendocrine tumor; SCLC, small cell lung cancer.

^*a*^MTT treatment allowed the initial occult carcinoid tumors to be located in 11 patients during further follow-up.

^*b*^Patient 32 received the triple therapy of mitotane, metyrapone, and ketoconazole.

^*c*^Adverse effects were not reported individually in report by Donadille et al [10]. Overall, the most common side effects were digestive and neurological signs, which were observed in 15 (65%) and 6 (26%) patients, respectively.

The dose and therapeutic concentrations of MTT required for EAS-related hypercortisolism are suggested to be lower than those for adrenocortical carcinoma (14-20 mg/L). Only quite few EAS cases have reported the plasma concentration of MTT, and the mean value was 10.4 ± 6.5 mg/L (range, 2-15.5 mg/L), with a mean daily dose of 3.3 ± 1.2 g/d [10]. Similar dose and plasma concentrations were observed in patients with CD [8]. Measurement of plasma MTT concentration was not yet feasible in our hospital, but for short-term treatment with MTT, compared to plasma concentration of MTT, a decrease in cortisol levels might be more sensitive and help guide optimal dose titration.

Patients with EAS generally tolerate MTT well. The most common side effects were mild gastrointestinal and neurological symptoms, which occurred in 65% and 26% patients, respectively [10]. Tolerance is dependent not only on the dose, but also on the duration of treatment, and side effects are reversible when discontinuing the drug or reducing the dose [3, 10]. Severe hepatoxicity was reported in 2 cases that had complex cofounding factors, such as comedication use of ketoconazole and underlying virus infection or metabolic liver diseases [3, 10]. In our case, transaminases were mildly elevated initially, and remained stable during the MTT treatment. No other side effect was observed.

We presented here a rare pediatric case of EAS caused by thymic NET treated with preoperative short-term MTT monotherapy that quickly stabilized hypercortisolism-associated life-threatening complications without adverse effects, and allowed for safer curative surgery. Importantly, the time needed to control hypercortisolism was shorter than commonly assumed. Therefore, instead of an “add-on drug”, we highlighted a beneficial role of preoperative short-term MTT therapy in the management of severe hypercortisolism in patients with EAS, especially in countries and areas where other adrenal steroidogenesis inhibitors are not accessible.

## Learning Points

- NET is a rare but important cause among pediatric EAS patients.
- MTT as monotherapy is an option to control severe hypercortisolism, especially in countries and areas where other adrenal steroidogenesis inhibitors are not accessible.
- Monotherapy with MTT could rapidly control hypercortisolism in patients with thymic NET-EAS with good tolerance, and the dose is much lower than that in adrenal carcinoma.

## Data Availability

Data sharing is not applicable to this article as no datasets were generated or analyzed during the current study.

## Abbreviations

^18^F-FDG: ^18^F-fluorodeoxyglucose
ACTH: adrenocorticotropic hormone
CD: Cushing disease
CS: Cushing syndrome
CT: computed tomography
EAS: ectopic adrenocorticotropin syndrome
MTT: mitotane
NET: neuroendocrine tumor
PET: positron emission tomography
RR: reference range
SUVmax: maximum standardized uptake value
UFC: urinary free cortisol
